# Improving identification of symptomatic cancer at primary care clinics: A predictive modeling analysis in Botswana

**DOI:** 10.1002/ijc.34178

**Published:** 2022-06-29

**Authors:** Kesaobaka Molebatsi, Hari S. Iyer, Racquel E. Kohler, Kemiso Gabegwe, Isaac Nkele, Bokang Rabasha, Kerapetse Botebele, Tomer Barak, Siamisang Balosang, Neo M. Tapela, Scott L. Dryden‐Peterson

**Affiliations:** ^1^ Department of Statistics University of Botswana Gaborone Botswana; ^2^ Botswana‐Harvard AIDS Institute Partnership Gaborone Botswana; ^3^ Department of Epidemiology Harvard T. H. Chan School of Public Health Boston Massachusetts USA; ^4^ Division of Population Sciences Dana‐Farber Cancer Institute Boston Massachusetts USA; ^5^ Department of Health Behavior, Society and Policy, Rutgers School of Public Health Piscataway New Jersey USA; ^6^ Department of Social and Behavioral Sciences Harvard T. H. Chan School of Public Health Boston Massachusetts USA; ^7^ Department of Medicine Beth Israel Deaconess Medical Center Boston Massachusetts USA; ^8^ Nuffield Department of Population Health Oxford University Oxford UK; ^9^ Department of Medicine Harvard Medical School Boston Massachusetts USA; ^10^ Department of Infectious Diseases Brigham and Women's Hospital Boston Massachusetts USA

**Keywords:** predictive modeling, symptomatic cancer

## Abstract

In resource‐limited settings, augmenting primary care provider (PCP)‐based referrals with data‐derived algorithms could direct scarce resources towards those patients most likely to have a cancer diagnosis and benefit from early treatment. Using data from Botswana, we compared accuracy of predictions of probable cancer using different approaches for identifying symptomatic cancer at primary clinics. We followed cancer suspects until they entered specialized care for cancer treatment (following pathologically confirmed diagnosis), exited from the study following noncancer diagnosis, or died. Routine symptom and demographic data included baseline cancer probability assessed by the primary care provider (low, intermediate, high), age, sex, performance status, baseline cancer probability by study physician, predominant symptom (lump, bleeding, pain or other) and HIV status. Logistic regression with 10‐fold cross‐validation was used to evaluate classification by different sets of predictors: (1) PCPs, (2) Algorithm‐only, (3) External specialist physician review and (4) Primary clinician augmented by algorithm. Classification accuracy was assessed using c‐statistics, sensitivity and specificity. Six hundred and twenty‐three adult cancer suspects with complete data were retained, of whom 166 (27%) were diagnosed with cancer. Models using PCP augmented by algorithm (c‐statistic: 77.2%, 95% CI: 73.4%, 81.0%) and external study physician assessment (77.6%, 95% CI: 73.6%, 81.7%) performed better than algorithm‐only (74.9%, 95% CI: 71.0%, 78.9%) and PCP initial assessment (62.8%, 95% CI: 57.9%, 67.7%) in correctly classifying suspected cancer patients. Sensitivity and specificity statistics from models combining PCP classifications and routine data were comparable to physicians, suggesting that incorporating data‐driven algorithms into referral systems could improve efficiency.

## What's new?

In Africa, progress in the delivery of cancer care remains overshadowed by the persistence of barriers in the referral process from primary care to specialized oncology care. As a consequence, many Africans continue to suffer delays in cancer diagnosis and treatment. The present study assessed the possibility of using data‐driven algorithms to augment primary care provider (PCP)‐based referrals for suspected cancer cases. Models show that initial PCP assessment augmented by routine data facilitates correct classification of suspected cancer cases. The findings suggest that risk scores derived from data‐driven models could improve PCP assessments of probable cancer, particularly in low‐resource settings.

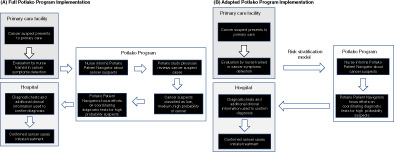

**Abbreviations**
AUC: area under the curve
ECOG: eastern cooperative oncology group
PCP: primary care provider
ROC: receiver operating curve

## 1 INTRODUCTION

Cancer patients in sub‐Saharan Africa experience some of the highest case fatality rates globally.^1^ The annual burden of cancer in the region is expected to increase to 1.27 million new cases and 1 million deaths from cancer by 2030.^2^ African governments have made important progress in strengthening cancer care delivery through task‐shifting, lower medicine costs, novel equipment and diagnostics and investments in radiotherapy and surgical centers.^3^, ^4^, ^5^, ^6^, ^7^, ^8^, ^9^, ^10^ Despite these achievements, many Africans still experience geographic and financial barriers that prevent them from accessing timely cancer treatment.^11^, ^12^, ^13^ Cancer programs often lack the appropriate infrastructure, medicines and equipment and trained staff needed to deliver high quality cancer care.^9^, ^14^, ^15^, ^16^ Improving the process of referral from primary to specialized oncology care centers through systems‐level interventions is critical for ensuring timely access to treatment.^13^, ^14^, ^17^, ^18^

Botswana, a middle‐income country in sub‐Saharan Africa, has a national cancer control program that focuses on integrating cancer diagnosis and treatment services into existing health care programs.^18^, ^19^, ^20^ Following successes in providing universal antiretroviral therapy for patients infected with human immunodeficiency virus (HIV),^21^, ^22^, ^23^, ^24^, ^25^ Botswana implemented national cervical cancer screening program starting in 2012. Free treatment is available to all citizens with cancer.^26^ However, delays in initiating treatment—sometimes as long as 7 months (IQR: 3.6 to 13.9) from initial presentation with cancer symptom^27^—contribute to over half of patients diagnosed with advanced disease.^27^

A key barrier to timely treatment is low cancer literacy among health providers whose training and experience has historically focused on maternal‐child health and infectious disease.^19^ We hypothesized that data‐driven algorithms could improve classification of patients at increased risk of having cancer among those identified by a primary care provider (PCP) as a cancer suspect. Potentially, diagnostic evaluation in high‐risk cancer suspects could be prioritized with improved access to specialist clinicians, scarce services (eg, cross‐sectional imaging, invasive biopsies and endoscopy) and expedited pathology review. Augmenting PCP referrals of patients suspected to have cancer (“cancer suspects”) with data‐driven algorithms could shorten time to treatment initiation and alleviate burden on limited numbers of trained oncologists.^28^, ^29^ Given that labor contributes 20% to oncology programmatic costs in African settings, efficiency gains accrued by task‐shifting could lead to substantial cost savings.^30^ Algorithmic predictions could enable more accurate PCP referrals of high‐risk patients for further examination by oncology care providers, and direct noncancer suspects to more appropriate services. Similar approaches have improved targeting of treatment to HIV‐positive individuals in African settings.^31^, ^32^, ^33^

Our study aimed to assess the potential effectiveness of data‐driven clinical decision‐making aids to support PCP referrals of cancer suspects in low‐resource settings. We compared classification of cancer suspects for whom ultimate cancer diagnosis was available across models using (1) PCP, (2) algorithm‐only, (3) external specialist physician review and (4) PCP augmented by algorithm.

## 2 METHODS

### 2.1 Study population and enrollment procedures (Potlako, CliicalTrials no: NCT02752061 & ID: BHP078)

**FIGURE 1:**
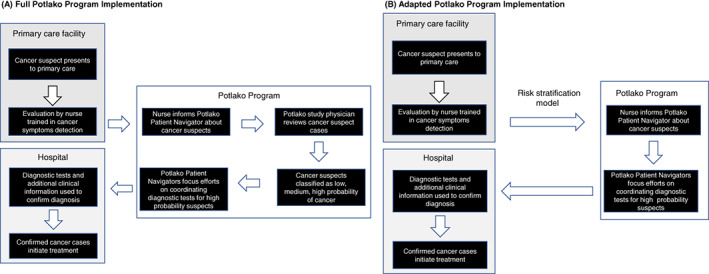
Alternative approaches for primary care referrals of cancer suspects under (A) full Potlako program implementation and (B) adapted implementation using data augmented risk stratification [Color figure can be viewed at wileyonlinelibrary.com]

In 2016, the Botswana‐Harvard AIDS Institute Partnership and Botswana's Ministry of Health, initiated the *Potlako* (“hurry” in Setswana) study intended to reduce delays in accessing oncology care and subsequently the stage at presentation among cancer patients.^19^ This health systems intervention had three components: (1) training primary care nurses or PCPs on common cancer‐related signs and symptoms, referral and principles of early cancer diagnosis, (2) patient navigators to guide patients through the referral process and liaise between oncology care providers and patients to ensure timely visits and (3) limited financial support to remove transportation barriers to accessing oncology care. As part of the intervention, PCPs received training in cancer symptom detection, diagnostic techniques used in oncology, and cancer epidemiology.^19^ Trained PCPs were responsible for documenting cancer suspects in clinic‐based register or communicating (by phone or in person) with *Potlako* patient navigators when a suspected cancer case needed referral. Study physicians (nononcologists) oversaw the process for referral and directed navigators to focus on patients who were deemed highly likely to have cancer. Specific tasks completed by *Potlako* personnel to confirm cancer diagnosis are outlined in Figure 1A.

Male and female cancer suspects presenting to primary care facilities in the rural Kweneng East District (estimated catchment population: 200 000) between April 2016 and June 2020 were enrolled in the study. Participants had to be 18 years or older, a resident of Kweneng East, not incarcerated, and not currently pregnant to be eligible for the study. PCPs, many of whom had received specialized training in detection of cancer symptoms, documented cancer suspects in a clinic‐based register and communicated these to study team either by phone or in person. Patients were followed from index facility visit until cancer diagnosis or exit from program due to exclusion of cancer, death prior to diagnosis or request to be removed. There were 952 patients with suspected cancer who met eligibility criteria, completed the study, and were assigned an exit outcome. We excluded participants who were missing demographic or clinical information at baseline.

### 2.2 Measures

As part of the *Potlako* intervention, PCPs and patient navigators collected data on demographic characteristics and symptoms from a cohort of cancer suspects. All health facilities in the participating health district received a *Potlako* register to facilitate follow‐up of patients identified as requiring evaluation as a cancer suspect. At the time of recording, PCPs recorded presenting symptoms, age, sex, HIV status, and the PCP's assessment of cancer probability (low, medium and high). Subsequently, *Potlako navigators* called patients to collect further demographic, clinical and contact details. Navigators were responsible for linking patients to care and coordinating care between hospital oncology services and laboratories. After the initial call, navigators shared details on all new suspected cases with *Potlako* specialist physicians on a weekly basis.

Cancer probability was assessed independently by study physicians and PCPs. PCPs received a standardized training in detection of presenting symptoms of common cancers in this population.^19^ Study physicians reviewed suspected cancer cases' demographics and clinical laboratory and pathology assessments to assign cancer probability. Using their clinical judgment and available information (including the PCP's assessment of cancer likelihood), external specialist physicians then assigned their own assessment of probability of cancer: low (noncancer causes of the presenting symptoms is more likely), medium (cancer is among the leading causes of presenting symptoms) and high (cancer is the leading cause of presenting symptoms). The same two physicians (NT and SDP) performed assessments throughout to ensure consistency.

We compared four sets of predictor measures commonly used by PCPs and physicians to stratify suspected cancer cases based on risk. Different predictor sets represent potential approaches to augment a PCP's decision to prioritize a patient for further cancer evaluation. Predictor set (1) included the PCP's own initial classification of cancer probability modeled as a categorical variable (low, medium or high). Predictor set (2) included routinely collected demographic characteristics and presenting symptoms (patient age and sex, presence, or absence of mass/lump, ECOG score and HIV status), which we refer to hereafter as “algorithm only.” We chose to include these factors because these elements are or could reasonably be routinely collected as part of clinical care. In the *Potlako* study, external specialist physicians (Authors: SDP, NMT) also reviewed summarized information from cancer suspects at time of initial presenting and provided their own assessment to direct patient navigators towards those cancer suspects deemed as having high risk of cancer. External specialist physicians also assigned probability of cancer as a categorical variable (high, medium and low). While this physician review may not be uniformly available in primary care settings in low‐resource contexts, we felt this would represent an “ideal” clinical workflow and so was included as predictor set (3). Finally, in predictor set (4) we chose to assess a model including predictor sets (1) and (2), representing augmentation of the PCP's assessment with routinely collected demographic characteristics and presenting symptoms in the absence of a specialist physician. We refer to predictor set (4) as “PCP augmented by algorithm”. The adopted Potlako program is outlined in Figure 1B.

### 2.3 Statistical analysis

We summarized participant demographic and clinical characteristics using frequencies and percentages for categorical variables and means and standard deviations for continuous variables. Chi‐square tests for independence and Fisher's exact tests were used to assess significant differences between categorical variables, while Wilcoxon rank sum tests were used to assess differences between continuous variables. We separately fit logistic regression models using each of the four predictor sets and estimated predicted probabilities of final cancer diagnosis (1 = probable or confirmed cancer, 0 = other). For Model 2 (Routine demographics and symptoms), we adjusted for age (quadratic), sex, performance status (categorical), pain score (categorical), HIV status (binary) and lump symptom (binary) as defined above. For Model 4 (PCP + Routine demographics and symptoms), we adjusted for the covariates above, along with intake referring clinician. We checked for model calibration using the Hosmer‐Lemeshow test. To correct for over‐optimistic model fit, we applied 10‐fold cross validation before reporting measures of predictive model performance. This procedure involves splitting the full dataset into 10 samples with observations chosen at random. Training is implemented in nine samples, with the remaining set held out for testing. This process is repeated 10 times (leaving a different set out each time), and the results are summarized across the 10 samples.^34^

To assess discrimination of our regression models containing each set of predictors, we reported c‐statistics and plotted receiver operating curves (ROC). Since costs of both false positive and negative classifications should be considered when selecting a decision‐making algorithm, we reported sensitivity, specificity, positive predictive value and negative predictive value. Since the predicted probabilities estimated from logistic regression models provide a continuous rather than binary set of values, we chose three thresholds for classifying a patient as having cancer based on whether their modeled predicted probability was ≥0.3, 0.4 or 0.5. Lower classification thresholds favoring higher sensitivity (correct classification of true cancer cases) and higher thresholds favoring higher specificity (correct classification of noncancer cases). As a sensitivity analysis, we compared predictors identified above to those derived from a data‐driven covariate selection approach using least absolute shrinkage and selection operator (LASSO).^35^ Results from LASSO, which also account for possible collinearity between predictors, did not differ from those obtained from our main analysis and so we report results from our main models.

We then estimated the total number of participants who would be classified as having low, moderate or high probability under each predictor set to determine how many total patients would be impacted by adopting the data‐driven algorithm. To generate low, moderate and high probabilities from our data‐driven models, we generated tertiles based on the distribution of predicted probabilities for predictor sets (2) and (4). All analyses were done using SAS (Version 9.4; SAS Institute, Cary, North Carolina).

## 3 RESULTS

### 3.1 Cohort characteristics

A total of 623 (65%) cancer suspects participating in the *Potlako* study with complete data were analyzed (Table 1). Participants who were excluded (n = 329, 35%) were of similar age, similarly likely to present with bleeding symptoms, and of those ultimately diagnosed with cancers, had similar patterns of final diagnoses. However, excluded patients were less likely to be HIV negative, more likely to be female and present with a lump, and less likely to have suspected breast or cervical cancer (Table S1). Of the 623, 166 (27%) of study participants were confirmed to have cancer. Confirmed cancer patients were older (median age: 59 vs 42 years, *P* < .0001), less likely to be female (n = 93, 56% vs n = 361, 79%, *P* < .0001) and more likely to be living with HIV (n = 67, 40% vs n = 119, 26%, *P* = .0010) compared with noncancer patients. PCPs classified 119 (21%), 368 (64%) and 89 (15%) of cancer suspects as having low, moderate and high cancer probability, respectively. PCPs classified 51 (32%) of cancer suspects in whom cancer was eventually confirmed as having high probability of cancer, and 109 (26%) of cases who did not go on to receive a cancer diagnosis as having low probability of cancer. In contrast, study physicians classified 81 (40%) of cases eventually confirmed as having cancer as having high probability, and 236 (52%) of cancer suspects who did not have cancer as having low probability.

**TABLE 1:** Demographic and clinical characteristics of *Potlako* study participants, Botswana, 2016 to 2020

	Confirmed cancer		Total	*P* ^a^
	No	Yes		
N (%)	457 (73)	166 (27)	623	
Age^b^	42 [29, 58]	59 [46, 71]	48 [33, 63]	<.0001^c^
Female	361 (79)	93 (56)	454 (73)	<.0001
What was patient's last HIV test result?				.0010^d^
Positive	119 (26)	67 (40)	186 (30)	
Negative	325 (71)	98 (59)	423 (68)	
Unknown	13 (3)	1 (1)	14 (2)	
How strong is PCP's suspicion for cancer?				<.0001
Low	109 (26)	10 (6)	119 (21)	
Moderate	269 (65)	99 (62)	368 (64)	
High	38 (9)	51 (32)	89 (15)	
*Potlako* physician cancer probability (baseline)				<.0001
Low	236 (52)	9 (5)	245 (39)	
Moderate	185 (40)	76 (46)	261 (42)	
High	36 (8)	81 (49)	117 (19)	
Symptoms				
Bleeding	51 (11)	28 (17)	79 (13)	.058
Lump	186 (41)	34 (20)	220 (35)	<.0001
Suspected cancer				
Breast	208 (46)	29 (17)	237 (38)	<.0001
Cervix	100 (22)	32 (19)	132 (21)	.48
Performance status (ECOG)				<.0001^d^
0	255 (56)	65 (39)	320 (51)	
1	157 (34)	56 (34)	213 (34)	
2	28 (6)	24 (14)	52 (8)	
3	13 (3)	13 (8)	26 (4)	
4	4 (1)	8 (5)	12 (2)	
Pain score				.016^d^
0	178 (39)	43 (26)	221 (35)	
1	142 (31)	50 (30)	192 (31)	
2	74 (16)	40 (24)	114 (18)	
3	42 (9)	23 (14)	65 (10)	
4	14 (3)	6 (4)	20 (3)	
5	7 (2)	4 (2)	11 (2)	
Final cancer diagnosis (Missing)			457 (73)	
Cervical cancer		31 (19)	31 (5)	
Breast cancer		30 (18)	30 (5)	
Esophageal cancer		14 (8)	14 (2)	
Kaposi's sarcoma		9 (5)	9 (1)	
Prostate cancer		14 (8)	14 (2)	
Other		46 (28)	46 (7)	

^a^ IQR, Chi‐square test for independence.

^b^ Median.

^c^ Wilcoxon rank sum test.

^d^ Fisher's exact test.

### 3.2 Symptoms and diagnoses

Common symptoms among cancer suspects included lumps (n = 220, 35%) and bleeding (n = 79, 13%). Cancer suspects who were eventually diagnosed with malignancy were more likely to report bleeding (n = 28, 17%) vs lump (n = 34, 20%). The most common suspected cancers were of the breast (n = 237, 38%) and cervix (n = 132, 21%). Among cancer suspects eventually diagnosed with cancer, the most diagnoses were of the cervix (n = 31, 19%), breast (n = 30, 18%), esophagus (n = 14, 8%), Kaposi sarcoma (n = 9, 5%) and prostate (n = 14, 8%), and other less common cancers (n = 46, 28%).

### 3.3 Classification of cancer suspects by predictor sets

Results of classifications under the predictive probability thresholds and different predictor sets revealed optimal trade‐offs between sensitivity and specificity when using a 0.4 threshold (Table 2). With respect to sensitivity, predictor set (4), PCP with data augmentation, (50%) outperformed predictor set (3) (49%), (2) (48%) and (1) (31%) in correctly classifying cancer suspects who went on to have confirmed cancer. Regarding specificity, or correctly classifying cancer suspects who ultimately did not have cancer, predictor set 1, PCP initial assessment, (91%) outperformed predictor set 4 (83%), predictor set 2 (81%) and predictor set 3 (80%). Predictor set (3) yielded the highest positive predictive value (69%), followed by (1) (55%), (3) (51%) and (2) (48%). Predictor set (3) yielded the highest negative predictive value of (83%), followed by model 4 (82%), model 2 (81%) and model 1 (78%). Comparisons of classification indicators across other cut points (30%, 50%) demonstrate that sensitivity is optimized at lower thresholds, whereas specificity is optimized at higher thresholds. Losses in sensitivity with increasing thresholds occur more quickly than gains for other indicators.

**TABLE 2:** Sensitivity and specificity for logistic regression models to predict probability of cancer using different covariate sets, Botswana Potlako program for early detection of cancer, 2016 to 2020

	Cutpoint for thresholds											
	30%				40%				50%			
Proportion (95% CI)	Sensitivity	Specificity	PPV	NPV	Sensitivity	Specificity	PPV	NPV	Sensitivity	Specificity	PPV	NPV
Model 1	31 (24, 38)	91 (88, 93)	55 (45, 65)	78 (75, 82)	31 (24, 38)	91 (88, 93)	55 (45, 65)	78 (75, 82)	31 (24, 38)	91 (88, 93)	55 (45, 65)	78 (75, 82)
Model 2^a^	67 (60, 74)	68 (64, 73)	44 (37, 50)	85 (81, 89)	48 (40, 55)	81 (78, 85)	48 (41, 56)	81 (77, 85)	25 (19, 32)	90 (87, 93)	48 (38, 58)	77 (73, 80)
Model 3^b^	55 (48, 63)	70 (66, 75)	53 (45, 60)	83 (80, 86)	49 (41, 56)	80 (76, 84)	69 (61, 78)	83 (80, 86)	49 (41, 56)	89 (86, 92)	84 (80, 87)	83 (80, 86)
Model 4	68 (61, 75)	72 (68, 76)	47 (41, 53)	86 (83, 90)	50 (42, 58)	83 (79, 86)	51 (43, 58)	82 (78, 85)	29 (22, 36)	90 (87, 92)	50 (40, 60)	78 (74, 81)

*Note*: Model 1: PCP; Model 2: Routine demographics and symptoms; Model 3: Potlako physician and Model 4: PCP + Routine demographics and symptoms.

Abbreviations: NPV, negative predictive value; PCP, primary care provider; PPV, positive predictive value.

^a^ Adjusted for age (quadratic), sex, performance status (categorical), pain score (categorical), HIV status (binary) and lump symptom.

^b^ Adjusted for age (quadratic), sex, performance status (categorical), pain score (categorical), HIV status (binary), intake referring clinician (categorical) and lump symptom.

**FIGURE 2:**
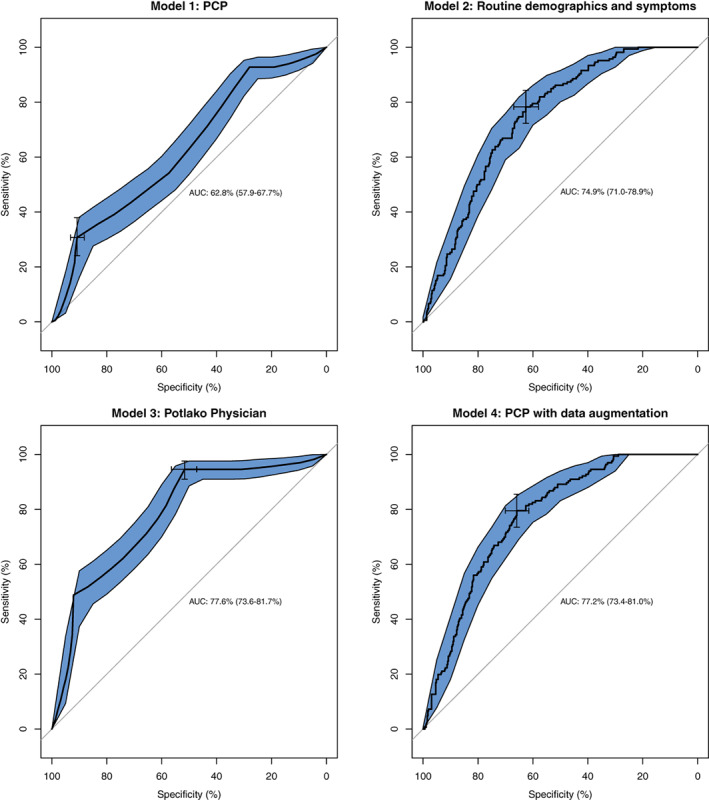
Area under curve and 95% confidence interval for models to predict cancer among all suspected cases (n = 623) using primary care providers, routine demographic and symptoms, Potlako physician and primary care providers + routine demographic and symptoms [Color figure can be viewed at wileyonlinelibrary.com]

ROC showed similar discrimination for predictor sets (3) and (4) (Figure 2). The area under the curve (AUC) for predictor set (3) was the highest (77.6%, 95% CI: 73.6%, 81.7%), followed by predictor set (4) (77.2%, 95% CI: 73.4%, 81.0%), predictor set (2) (74.9%, 95% CI: 71.0%, 78.9%) and predictor set (1) (62.8%, 95% CI: 57.9%, 67.7%). AUC was significantly higher for predictor set (2) compared with (1) (*P* < .0001), and for (4) compared with (1) (*P* < .0001). There was no significant difference between AUC for predictor set (3) compared with (4) (*P* = .87).

To determine the number of cancer suspects affected by implementing different classification algorithms, we calculated the proportion of those diagnosed with confirmed cancer and those not diagnosed with cancer under each predictor set (Table 3). Of 166 total cancer suspects who were eventually diagnosed with cancer, compared with predictor set (1), predictor set (4) augmenting PCP initial assignment with data would result in an increase of 50 participants being classified as having high probability of cancer. Of 457 cancer suspects who did not have cancer, predictor set (4) would result in 65 additional patients classified as having low probability of cancer, although there would also be an increase of 65 patients incorrectly classified as having high probability of cancer.

**TABLE 3:** Numbers of cancer suspects classified as low, moderate and high cancer probability by different predictor sets in the Botswana Potlako program for early detection of cancer

	Confirmed cancer		Total
	No	Yes	
	n (%)	n (%)	
Model 1			
Low	128 (28)	12 (7)	140 (22)
Moderate	287 (63)	103 (62)	390 (63)
High	42 (9)	51 (31)	93 (15)
Model 2^a^			
Low (<0.155)	193 (42)	14 (8)	207 (33)
Moderate (0.156 to <0.339)	153 (33)	55 (33)	208 (33)
High (>0.339)	111 (24)	97 (58)	208 (33)
Model 3^b^			
Low	236 (52)	9 (5)	245 (39)
Moderate	185 (40)	76 (46)	261 (42)
High	36 (8)	81 (49)	117 (19)
Model 4			
Low (<0.122)	193 (42)	14 (8)	207 (33)
Moderate (0.123 to <0.346)	157 (34)	51 (31)	208 (33)
High (≥0.350)	107 (23)	101 (61)	208 (33)
Total	457	166	623

*Note*: Model 1: PCP; Model 2: Routine demographics and symptoms; Model 3: Potlako physician; Model 4: PCP + Routine demographics and symptoms. For Models 2 and 4, low, moderate and high were generated using tertiles and the range of predicted probabilities falling in each category are provided.

^a^ Adjusted for age (quadratic), sex, performance status (categorical), pain score (categorical), HIV status (binary) and lump symptom.

^b^ Adjusted for age (quadratic), sex, performance status (categorical), pain score (categorical), HIV status (binary), intake referring clinician (categorical) and lump symptom.

## 4 DISCUSSION

In this study comparing different sets of predictors to estimate probability of cancer among cancer suspects in Botswana, we found that predictions derived from models with PCP augmented by routine data improved correct classification relative to the initial PCP's assessment alone. Predictions from models relying on PCP augmented by routine data led to discrimination and sensitivity metrics comparable to those from models relying on physician's initial assessment. In general, combining information from predictor sets yielded improved classification of noncancer cases, reflected by higher specificity and negative predictive values. These findings imply that PCPs could improve their assessments of probable cancer by using predictions from data driven algorithms that incorporate routine demographic and symptom data. Empowering PCPs to conduct these assessments would alleviate burden on physicians, who could then focus on patients most likely to have cancer.

Although risk scores have been developed for many diseases, including cardiovascular disease and breast cancer in white populations,^36^, ^37^ there have been few attempts to integrate risk scores into diagnostic and referral procedures for oncology care in sub‐Saharan Africa. In part, this may reflect lack of high‐quality data prior to entry into clinical care. One recent study demonstrated feasibility of an automated radiation planning assistant to be used in South Africa to support clinical staff in dosing for cancer treatment.^38^ A case‐control study in Nigeria using known breast cancer risk factors tested a novel Nigeria‐specific breast cancer prediction model yielding a c‐statistic of 0.70, comparable to those derived in African‐American populations.^39^ In contrast, there have been numerous examples of clinical prediction tools implemented in sub‐Saharan Africa to stratify HIV/AIDS patients by risk and improve treatment initiation and outcomes, combining regression coefficients with score‐based checklists to be used by providers.^31^, ^32^ Experiences from HIV suggest that it would be feasible to introduce checklist‐based tools to PCPs leveraging the predictive models presented here to facilitate appropriate referrals for specialized oncology care.

An important area for future research is tailoring risk scores to specific cancers based on the geographic region of interest. In our study, there was low prevalence of two common cancers in other sub‐Saharan African settings, lung and prostate. While population‐based estimates of smoking prevalence in Botswana are limited, occupational surveys in adult teachers reported a prevalence of 3.2%,^40^ and a more recent population‐based survey of adults reported a prevalence of 11.6%.^41^ The low prevalence of a major risk factor for lung cancer could explain the low rates of lung cancer identified in our study. For prostate cancer, there have been reports that HIV‐infected patients on ART experience lower rates of many common cancers including prostate than members of the general population.^42^ It is possible that HIV infection disrupts androgen metabolism and circulating hormone levels, but these remain hypotheses that warrant further study. Regardless, developing appropriate risk scores to support referrals must take the local cancer epidemiology into account.

High‐income countries with government‐sponsored health care systems are incentivized to ensure accuracy of cancer referrals because the public sector most control costs. In these settings, PCPs without specialist training serve as gatekeepers.^43^, ^44^, ^45^ In order to develop consensus statements and guidelines to guide referrals for specialized oncology care, experts review literature to identify nonspecific symptoms that could apply to a broad range of cancers based on Positive Predictive Value associated with each symptom.^43^ In our study, we were unable to develop cancer‐specific models due to limited sample size, but this could be explored in the future. Other African countries have begun to introduce cancer‐specific early detection programs. For example, in Rwanda, a district‐wide early detection program for breast cancer led to improvements in appropriate referrals for breast cancer, increased case detection and lower stage at diagnosis.^46^, ^47^

We were limited in the sociodemographic and clinical predictors that were included in our analysis, choosing to focus on elements that could be acquired through routine data collection. Studies of cancer patients using register‐based cohorts and prospective data collection have identified several factors that could be considered to improve model performance. These factors include distance from participants' residence to closest primary care facility, rurality, information about income and education and knowledge and perceptions of cancer treatment and outcomes.^27^, ^29^, ^48^, ^49^, ^50^, ^51^, ^52^, ^53^ Collection of participant's residential location (eg, village or town) could allow estimation of distance to care and improve model performance with little additional data collection burden. Given that infectious agents are a major contributor to cancer incidence in sub‐Saharan Africa, more detailed history of infectious diseases could also improve model performance.^54^, ^55^ In Botswana, the high burden of HIV and coverage of ART suggests that greater communication between providers in infectious disease clinics and cancer care could facilitate these data linkages.

This study had several strengths and limitations. Strengths of this study include prospective data collection within a cohort of cancer suspects with complete follow‐up, along with baseline assessments of cancer probability from PCPs and study physicians in a peri‐urban African setting. Our study was implemented in the context of an ongoing health systems intervention to reduce delays and stage at diagnosis in Botswana, which may not reflect current cancer care delivery practices in other parts of Botswana or other African countries. However, as all cancer care systems must eventually rely on referral from lower to higher levels of care within the health system, with inherent challenges of reaching rural, more socially vulnerable patients,^12^, ^13^ experiences from our study could inform efforts to expand cancer referral programs in other low‐income settings. Introducing the statistical models, we present here into routine clinical practice in Botswana would require simplifying model outputs. This could be done using a risk score card‐based approach or software application.^31^ Since different cancers involve different diagnostic procedures and symptoms, cancer‐specific models would likely yield improved model performance. However, due to the limited sample size, it was not possible to consider specific cancer types.

## 5 CONCLUSION

We found that data‐driven algorithms relying on PCP's initial assessment augmented by routine data led to improvements in classifying cancer suspects who had cancer in a peri‐urban population of Botswana. It is possible that data‐driven algorithms can complement clinical training of PCPs because algorithms will make predictions based on relationships between risk factors and cancer drawn from the target population. Use of these model‐derived risk scores to guide PCP referrals could alleviate burden on physicians in low‐resource settings. Including additional predictors such as residential address and infectious disease history, and careful consideration of costs of false positives compared with false negatives, could improve efficiency in patient referrals, leading to shorter times between symptom onset, diagnosis and treatment initiation among cancer patients in Botswana. In resource‐limited settings, augmenting clinical decision making with prediction models that leverage routinely collected data could lead to more efficient referral practices and improved outcomes for patients suffering from cancer.

## AUTHOR CONTRIBUTIONS

**Kesaobaka Molebatsi:** conceptualization, data curation, formal analysis, writing‐original draft. **Hari S. Iyer:** conceptualization, data curation, formal analysis, writing‐original draft. **Racquel E. Kohler:** conceptualization, Writing‐review and editing. **Kemiso Gabegwe:** writing‐review and editing, data curation. **Isaac Nkele:** writing‐review and editing, data curation. **Bokang Rabasha:** writing‐review and editing. **Kerapetse Botebele:** writing‐review and editing, data curation. **Tomer Barak:** writing‐review and editing. **Siamisang Balosang:** writing‐review and editing, data curation. **Neo M. Tapela:** supervision, project administration, writing‐review and editing. **Scott L. Dryden‐Peterson:** conceptualization, supervision, project administration, writing‐review and editing. The work reported in the paper has been performed by the authors, unless clearly specified in the text.

## FUNDING INFORMATION

Hari S. Iyer was supported by NIH T32 CA 009001 and Kesaobaka Molebatsi was supported by DEL‐15‐006 and 107752/Z/15/Z.

## CONFLICT OF INTEREST

The authors declare no conflict of interest.

## ETHICS STATEMENT

This study was reviewed and approved by the institutional review board at the Harvard T. H. Chan School of Public Health and the Botswana Ministry of Health. Written documentation of informed consent was obtained from all individual participants included in the study.

## Supporting information


**TABLE S1** Comparison of patient characteristics among participants in full Potlako sample to those retained for analysis

## Data Availability

Anonymised *Potlako* dataset and data analysis code are available from the corresponding author upon request, provided that all Institutional Review Board requirements are satisfied.
