# Supplementary material for: Improving identification of symptomatic cancer at primary care clinics: A predictive modeling analysis in Botswana
Source: Int J Cancer. 2022 Jun 29;151(10):1663–73. doi: 10.1002/ijc.34178 (PMC10286759; doi:10.1002/ijc.34178)
Supplement: Supplementary file 1 — TABLE S1 Comparison of patient characteristics among participants in full Potlako sample to those retained for analysis [file IJC-151-1663-s001.pdf]

Supplementary Material

Improving identification of symptomatic cancer at primary care clinics: A predictive modeling analysis in Botswana

Kesaobaka Molebatsi, Hari S. Iyer, Racquel E. Kohler, Kemiso Gabegwe, Isaac Nkele, Bokang Rabasha, Kerapetse Botebele, Tomer Barak, Siamisang Balosang, Neo M. Tapela, Scott L. Dryden-Peterson

Table of Contents

Supplementary Table 1. Comparison of patient characteristics among participants in full Potlako sample to those retained for analysis

Supplementary Table 1. Comparison of patient characteristics among participants in full Potlako sample to those retained for analysis

	Retained in Study		
	Yes	No	Total
**N (%)**	623 (65)	329 (35)	952 (100)
**Age***	48 (33, 63)	46 (34, 62)	47 (33, 63)
**Female**	454 (73)	259 (79)	713 (75)
**What was patient's last HIV test result?**			
Positive	186 (30)	108 (33)	294 (31)
Negative	423 (68)	194 (59)	617 (65)
Unknown	14 (2)	27 (8)	41 (4)
**How strong is PCP's suspicion for cancer?**			
Missing	-	269 (82)	269 (28)
Low	140 (22)	11 (3)	151 (16)
Moderate	390 (63)	37 (11)	427 (45)
High	93 (15)	12 (4)	105 (11)
***Potlako* physician cancer probability (baseline)**			
Missing	-	2 (1)	2 (0)
Low	245 (39)	138 (42)	383 (40)
Moderate	261 (42)	126 (38)	387 (41)
High	117 (19)	63 (19)	180 (19)
**Symptoms**			
Bleeding	79 (13)	40 (12)	119 (13)
Lump	220 (35)	145 (44)	365 (38)
**Suspected Cancer**			
Breast	237 (38)	70 (21)	307 (32)
Cervix	132 (21)	53 (16)	185 (19)
**Performance Status (ECOG)**			
Missing	-	247 (75)	247 (26)
0	320 (51)	46 (14)	366 (38)
1	213 (34)	27 (8)	240 (25)
2	52 (8)	2 (1)	54 (6)
3	26 (4)	2 (1)	28 (3)
4	12 (2)	2 (1)	14 (1)
**Pain score**			
Missing	-	213 (65)	213 (22)
0	221 (35)	42 (13)	263 (28)
1	192 (31)	25 (8)	217 (23)
2	114 (18)	21 (6)	135 (14)
3	65 (10)	18 (5)	83 (9)
4	20 (3)	5 (2)	25 (3)
5	11 (2)	5 (2)	16 (2)
**Final Cancer Diagnosis**	166 (100)	98 (100)	264 (100)
Cervical Cancer	31 (19)	23 (23)	54 (20)
Breast Cancer	30 (18)	21 (21)	51 (19)
Esophageal Cancer	14 (8)	6 (6)	20 (8)
Kaposi's sarcoma	9 (5)	7 (7)	16 (6)
Prostate Cancer	14 (8)	5 (5)	19 (7)
Other	46 (28)	21 (21)	67 (25)
